# Editorial

**Published:** 1891-07

**Authors:** 


### Editorial.

### THE NORTH CAROLINA STATE BOARD OF EXAMINATION.

Through the kindness of Dr. Russell Bellamy, of Wilmington, N. C., we have received a copy of the examination recently held in Asheville by the Medical Examining Board of that State. The examination showed that North Carolina wished to have only those physicians whose ability had been tested. It is with a feeling of regret that the same cannot be said of the State of Georgia. We have always been in favor of a State Medical Examining Board, and at no distant date our views will be given upon this subject. The examination held in North Carolina was very thorough and practical, and was by no means one which college boys would call a "snap." The examiners were thoroughly apace with the times, as many of their questions show. We give a few from the different subjects:

Chemistry:—What is ozone? Give some of its characteristics.

Physiology:—Give (a) reaction of gastric juice, (b) composition in full, (c) average daily amount excreted and changes affected in food in stomach.

Give histology of an intestinal villus, and describe in detail how chyle is removed from small intestines.

Surgery:—Classify burns and give reason for dividing into classes, (b) prognosis and treatment.

How would you recognize the difference between hip joint dislocation and fracture of the neck of the femur; give treatment.

Obstetrics:—Causes and dangers of delayed and precipitate labor. Indications and contraindications and dangers of ergot.

Anatomy:—What structures are devided in an amputation of the middle of upper one-third of forearm.

Practice:—Give causes of ascites.

From these few questions selected from the examination, the general character of the latter may be surmised. We are glad to see this elevation of medical standard in our sister State.

### HIGHER MEDICAL EDUCATION.

It is with pleasure that we note the tendency in the various medical schools to a more thorough instruction in all its branches by an increase in the number of courses required for graduation. Harvard has already adopted the four-year course, and a circular received from the University of Pennsylvania states the following:

At a meeting of the Board of Trustees of the University held May 21st, Dr. Pepper made an offer of $50,000 toward an endowment fund of $250,000, and of $1,000 annually towards a guarantee fund of $20,000 annually for five years, conditioned upon the establishment of an obligatory graded four-year course of medical study. This was accompanied by a communication from the Medical Faculty pledging themselves to carry out this proposal, and to enter upon the four-year course in September, 1893.

The thoroughness and rapid advances which are being made in every department of medicine, necessitates a more prolonged study. That the mind may not be afflicted with simply a "smattering" knowledge of the subjects, you must give it time to assimilate that which it has already received. The field of medicine is becoming broader every day. The tendency to specialism is still on the increase, due in a great measure to the fact that young men think it will require a shorter time to become acquainted with one branch. Let the medical schools keep the iron hot, and inspire nobler ideas of the study of medicine.

### INTERNATIONAL CONGRESS OF HYGIENE AND DEMOGRAPHY.

The above Congress will assemble in London on August 10th, and continue to the 18th. The word demography comes from two Greeks words, damos, people and graphien, to write, meaning "the study of life conditions of communities from a statistical point of view." The Congress is expected to have delegates from all the civilized countries of the world. The

United States will be represented by Dr. J. S. Billings, the director of the hygienic department in the University of Pennsylvania. The meeting is to be presided over by H. R. H., the Prince of Wales. The Congress bids fair to be one of much interest and importance.

---

We are indebted to the Dios Chemical Company, for a very fine chart of the uterus and appendages. They offer it free to all physicians who apply for it.

---

We desire to call attention to our new department—"The Prescription Department." We wish all physicians who have any prescriptions that they have found of special value would send them to us and we will give them credit for the same.

---

Dr. Russell Bellamy, of Wilmington, N. C., won the "Appleton Prize" in Asheville last month, he having stood the best examination out of eighty applicants for license before the Board.

---

On the night of June the 10th, Dr. Wm. Perrin Nicholson was united in marriage to Miss Carolyn Clayton Crane. Miss Crane was one of the reigning belles of the Gate City, and a woman greatly admired Dr. Nicholson, as a surgeon and one of the editors of THE RECORD, possesses a justly deserved reputation. We extend him our best wishes.

---

A NEW FOOD.—Lacto-Cereal Food is a new product recently put on the market by Reed & Carnrick, of New York.

It is prepared from milk, cereals and fruit, and is not only palatable but highly nutritious and easily digested.

Great progress has been made in recent years in making foods to meet various indications. The Lacto-Cereal Food is especially prepared for invalids, the aged, and for convalescents who need a palatable, digestible, perfect food for building up waste tissues at the least possible expense of digestive effort.—*Dietetic Gazette.*

